# Psychological Harm in High‐Fidelity Simulation: A Concept Analysis

**DOI:** 10.1155/nrp/2002234

**Published:** 2026-07-08

**Authors:** Mohamed Toufic El Hussein, Dawson S. Sheehan

**Affiliations:** ^1^ Faculty of Health, Community and Education, School of Nursing and Midwifery, Mount Royal University, Calgary, Alberta, Canada, mtroyal.ca; ^2^ Faculty of Nursing, University of Alberta, Edmonton, Alberta, Canada, ualberta.ca; ^3^ Medical Cardiology, Coronary Care Unit, Rockyview General Hospital, Calgary, Alberta, Canada; ^4^ Department of Cardiac Sciences, Cumming School of Medicine, University of Calgary, Calgary, Alberta, Canada, ucalgary.ca

## Abstract

**Aim:**

To clarify and define the concept of psychological harm in high‐fidelity simulation.

**Background:**

High‐fidelity simulation is widely used in undergraduate nursing education to promote clinical competence and decision‐making. While often beneficial, emotionally intense or poorly supported simulation experiences can result in psychological harm, an underrecognized phenomenon that may disrupt learning, self‐efficacy, and professional development. Current literature lacks conceptual clarity to distinguish psychological harm from expected simulation‐related stress.

**Design:**

Concept analysis.

**Methodology:**

A concept analysis was conducted using the method developed by Walker and Avant to explore psychological harm in the context of high‐fidelity simulation.

**Data Sources:**

Peer‐reviewed literature published between 2010 and 2025 was retrieved from MEDLINE, CINAHL, PsycINFO, and TRIP Pro. Inclusion criteria focused on articles that discussed psychological or emotional responses to high‐fidelity simulation in healthcare education. Empirical peer‐reviewed literature informed the identification of defining attributes, antecedents, and consequences. Practice‐oriented documents and standards were reviewed to contextualize disciplinary usage; studies focused solely on technical skill acquisition were excluded.

**Results:**

Three defining attributes were identified: (1) absence of psychological safety, (2) emotional disruption exceeding expected learning stress, and (3) sustained emotional impact beyond the simulation experience. Antecedents included emotionally intense content, insufficient preparation, unskilled facilitation, and performance‐oriented learning environments. Consequences included diminished self‐confidence, avoidance of future simulation, and impaired transfer of learning to clinical practice. Recent qualitative findings revealed that harm is often internalized and shaped by relational dynamics with simulationists and peers.

**Conclusion:**

Psychological harm in high‐fidelity simulation is a multidimensional, learner‐dependent experience that can hinder both academic and professional development. This concept analysis offers a foundational definition and theoretical structure to support future research, education, and policy related to learner well‐being in simulation.

**Reporting Method**
 The EQUATOR guidelines for PRISMA were met.

## 1. Introduction

Simulation‐based education has become an essential component of healthcare training, providing undergraduate nursing students with a safe environment to develop clinical skills, critical thinking, and decision‐making abilities [1–4]. High‐fidelity simulation (HFS), as defined in the Healthcare Simulation Dictionary [5], refers to simulation‐based learning experiences that incorporate high levels of physical, conceptual, and psychological fidelity to support immersive learning. This type of simulation leverages the use of computerized mannequins and real‐time physiological responses to improve clinical competence and preparedness among nursing students [6, 7]. Additionally, HFS introduces collaborative learning into nursing education, which is foundational for effective multidisciplinary teamwork in future clinical practice and may enhance learners’ self‐awareness, empathy, and patient‐centered care [8, 9]. Within simulation‐based education, facilitators, commonly referred to as simulationists, play a central role in shaping learner experience and maintaining psychological safety throughout prebriefing, facilitation, and debriefing [10]. While simulation is generally viewed as a positive pedagogical tool [6, 7, 9, 11, 12], concerns regarding psychological harm have emerged [13, 14]. Psychological harm during simulation can manifest as emotional distress [15], anxiety [16, 17], decreased self‐efficacy [18], or trauma [19, 20], potentially hindering the learning outcomes and professional development of undergraduate nursing students [21].

The concept of psychological harm in simulation remains poorly defined in the literature, often mistaken for stress or discomfort, which are elements that may be necessary for learning but do not necessarily equate to harm [22, 23]. As such, the aim of this concept analysis, using the framework outlined by Walker and Avant [24], is to clarify the meaning of psychological harm in HFS. We will also identify the uses, defining attributes, antecedents, consequences, and empirical referents of psychological harm. For the purposes of this analysis, psychological harm is provisionally understood as an intense or sustained emotional disruption arising from simulation‐based learning that exceeds expected educational stress and interferes with a learner’s ability to engage, reflect, or feel psychologically safe [5, 25, 26]. This working definition serves as an initial anchor for inquiry and is refined through the concept analysis process.

## 2. Background

According to the Healthcare Simulation Standards of Best Practice (HSSOBP), psychological safety is a state in which learners feel secure to engage, make mistakes, and express themselves without fear of humiliation or retribution. Similarly, the Healthcare Simulation Dictionary describes psychological safety as a learning environment in which individuals feel free to ask questions, voice concerns, and make mistakes without fear of embarrassment or negative consequences [5]. However, the Dictionary does not define psychological harm, underscoring the need for conceptual clarity on when learner distress moves beyond normal or adaptive stress responses.

Within the broader literature, psychological harm has been described as the enduring cognitive or emotional distress [27] resulting from the simulation experience [21]. In simulation‐based learning experiences, the facilitator’s actions and communication set the emotional tone and serve as the primary safeguard against psychological harm. When these conditions are absent, learners may experience cognitive overload, shame, or distress that exceeds the normal and expected stress of learning. While psychological safety supports self‐efficacy and effective learning [28, 29], harm may occur when the environment elicits unresolved emotions such as shame or anxiety [16, 17] that extend beyond the learning context [30]. Recent evidence shows that violations of psychological safety in simulation, often stemming from educator incivility, lack of training, or disregard for standards, can result in fear, avoidance, and long‐term emotional distress among students [31]. These findings underscore that psychological harm is not inherent to simulation itself but arises when facilitation deviates from established best practices. Trauma‐informed facilitation, transparent expectations, and empathetic debriefing are therefore essential to protecting learner well‐being and aligning with best‐practice standards in simulation education [20, 32].

While HFS can evoke a range of emotional responses [33], not all negative feelings constitute psychological harm [34]. Elements such as stress, discomfort, or performance pressure are often inherent in HFS and are widely recognized as normal [35], or even beneficial aspects of experiential learning [22]. When appropriately managed within a psychologically safe environment, these responses can enhance critical thinking and improve resilience among learners [20, 29]. However, some of these expected stressors may be misinterpreted as indicators of psychological harm [13, 33], which may obscure the true nature of this concept.

HFS often replicates emotionally intense clinical scenarios to prepare undergraduate students for the realities of nursing practice [9]. Situations involving patient death, pediatric emergencies, code blue events, or terminal diagnoses are often used to develop clinical competence and emotional preparedness [6, 36]. While these experiences are pedagogically valuable [36, 37], they can evoke strong emotional reactions, particularly in novice learners who may lack the necessary coping strategies or prior exposure to adequately contextualize the simulation experience [32, 33, 38]. When delivered without adequate psychological support, these HFSs have the potential to overwhelm learners [32].

Psychological harm does not solely arise from the context of the simulation but rather from how the scenario is experienced and processed by the learner [39]. Emotional stressors introduced by HFS can be perceived variably [35], with some students reporting that stress enhances their learning and prepares them for real‐world challenges [17, 22], while others experience stress as excessive or harmful [16, 19]. Additionally, facilitators may unintentionally escalate the emotional risk of simulation by failing to assess individual learner readiness or provide trauma‐informed debriefing [20]. Although many simulation studies advocate for psychological safety [20, 29, 40], far fewer explore the nature, impact, or prevention of psychological harm.

A literature review on the concept of psychological harm within simulation‐based education revealed various disciplinary uses and definitions. This ambiguity poses a challenge for simulationists and researchers when attempting to differentiate between healthy emotional responses to stress and genuine harm. Psychological harm is often vaguely referenced in the literature [26], with some authors suggesting that it encompasses negative emotional outcomes, such as anxiety [16, 17] or distress [15, 41], while others use it to describe trauma [19, 20]. However, these descriptors are highly varied and can overlap with experiences that are integral to the learning process [33]. It is important to recognize that stress exists on a continuum with certain levels enhancing learning while others may cross the threshold into psychological harm, leading to notable deterioration [42]. For instance, productive stress and psychological discomfort are frequently observed in simulation and can be powerful in promoting growth and fostering resilience among learners [22, 23]. In contrast, trauma can lead to emotional suppression or a maladaptive coping strategy among learners [19, 20], thus decreasing their self‐efficacy and participation in simulation [21, 26, 43].

Without conceptual clarity, simulationists lack the necessary tools to identify and mitigate psychological harm in simulation. This gap not only limits the development of emotionally safe learning environments but also risks normalizing distressing learner experiences, especially among undergraduate nursing students. As such, a structured concept analysis was warranted to explore and refine the meaning of psychological harm in HFS.

## 3. Methodology

This concept analysis was conducted using the eight‐step approach described by Walker and Avant [24], which provides a structured method for clarifying the attributes, antecedents, and consequences of complex concepts. This approach was chosen for its suitability in guiding conceptual inquiry within healthcare education and for its alignment with a constructivist understanding of how psychological harm is experienced by learners.

Walker and Avant’s [24] method comprises eight steps: (1) selecting the concept; (2) determining the aims or purposes of the analysis; (3) identifying all uses of the concept; (4) determining the defining attributes; (5) constructing a model case; (6) constructing additional cases (e.g., borderline, contrary, or related cases); (7) identifying antecedents and consequences; and (8) defining empirical referents. Together, these steps provide a comprehensive framework for systematically dissecting and clarifying the concept of interest.

Each step of Walker and Avant’s framework was operationalized through systematic engagement with the literature. Concept uses were identified through analysis of explicit and implicit definitions and descriptions across disciplines. Defining attributes were derived through iterative comparison of recurrent thematic patterns within the included studies. Antecedents and consequences were identified by examining conditions preceding reported distress and documented learner outcomes. Constructed cases were developed to illustrate the presence or absence of the defining attributes. Empirical referents were identified from measurable behavioral, physiological, and self‐reported indicators described in the literature.

To support the analysis, peer‐reviewed literature was reviewed and synthesized. Studies were included based on their relevance to the phenomenon rather than their methodological type. This approach was informed by principles of integrated synthesis [44], which prioritize conceptual relevance over methodological uniformity. Data extraction and coding were conducted by both authors. Initial codes were generated independently, followed by collaborative comparison and refinement. Discrepancies were resolved through discussion until consensus was reached. These themes informed the identification of defining attributes, model and contrary cases, antecedents, consequences, and empirical referents related to psychological harm in simulation.

Following data extraction, thematic analysis was conducted following Braun and Clarke’s [45] six‐phase framework: (1) familiarization with the data through repeated reading; (2) independent generation of initial codes; (3) collation of codes into potential themes; (4) iterative review and refinement of themes to ensure coherence; (5) definition and naming of themes, with distinctions drawn between defining attributes, antecedents, and consequences; and (6) synthesis and reporting of themes in alignment with Walker and Avant’s [24] concept analysis. Discrepancies were resolved through discussion until consensus was achieved.

The aim of this analysis is to advance conceptual clarity around psychological harm in HFS, particularly as it relates to the experiences of undergraduate nursing students, and to support the development of targeted educational strategies that mitigate harm while preserving the integrity of experiential learning. In addition to definitional clarity, the analysis was undertaken to address concerns about the potential misuse or dilution of the term psychological harm. Without clear boundaries, psychological harm may go unrecognized, leaving learners vulnerable to genuine distress or, conversely, may be overapplied in ways that mischaracterize the normal and necessary challenges of simulation‐based learning. Such misuse risks stifling educational innovation, limiting creativity in simulation design, or offering a rationale for disengagement or failure. This analysis therefore seeks to promote balanced, ethical, and creative simulation practices that are appropriately challenging, while supporting facilitators in navigating concerns about psychological harm in an evidence‐informed manner.

Given the inclusion of both empirical studies and practice‐oriented documents (e.g., standards and commentaries), the analysis required managing differing epistemological assumptions across scholarly and gray literature. Consistent with integrated synthesis approaches, conceptual relevance rather than methodological hierarchy guided inclusion. Gray literature was therefore used to contextualize simulation practice and clarify disciplinary usage, whereas empirical evidence informed the identification of defining attributes, antecedents, and consequences. This strategy ensured analytic rigor while preserving the breadth required to capture the conceptual complexity of psychological harm.

### 3.1. Data Sources

A comprehensive review of the existing literature was conducted using the MEDLINE, CINAHL, PsycINFO, and TRIP Pro databases. A sample search string included combinations of the following: (“psychological harm” OR “emotional distress” OR “psychological safety”) AND (“high‐fidelity simulation” OR “simulation‐based education”) AND (“nursing” OR “healthcare education”). Titles and abstracts were screened independently by both authors, followed by full‐text review for relevance. Reference lists of included articles were hand‐searched to identify additional relevant studies. Articles were included if they were peer‐reviewed, accessible in English, published between 2010 and 2025, and discussed psychological or emotional responses to HFS in nursing. Gray literature such as dissertations and conference presentations was excluded from empirical analysis; however, select practice‐oriented standards and commentaries were consulted to contextualize disciplinary usage. Studies that focused on technical skill acquisition without addressing the emotional or psychological outcomes from HFS were excluded from the review.

## 4. Results

**FIGURE 1:**
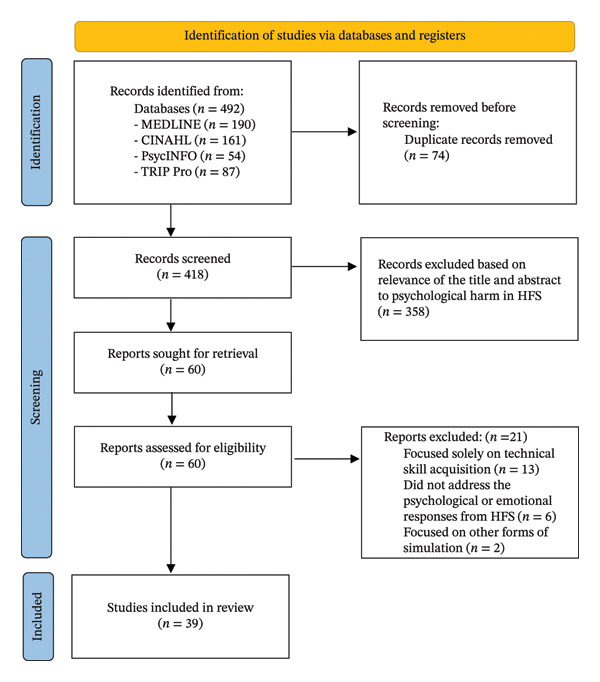
PRISMA flow diagram of the article selection process. PRISMA = Preferred Reporting Items for Systematic Reviews and Meta‐analyses.

The initial search yielded 492 articles. After the removal of 74 duplicates, the titles and abstracts of 418 articles were screened. Of these, 60 articles were selected for full‐text review based on preliminary relevance. Following the application of predetermined inclusion and exclusion criteria, 21 articles were excluded, resulting in a final sample of 39 peer‐reviewed publications included in the concept analysis (Figure 1 PRISMA flow diagram of the article selection process).

### 4.1. Concept Uses

This section corresponds to Step 3 of Walker and Avant’s [24] method, which involves identifying all uses of the concept across disciplines to establish its range of meanings.

The term psychological harm is broadly used across multiple disciplines to describe injury to a person’s emotional or mental well‐being [26]. It often overlaps with terms that are used to describe negative responses to simulated learning experiences, such as emotional distress [15, 27, 41], anxiety [16, 17], and a loss of psychological safety [46, 47]. While each of these constructs has a distinct theoretical background, they are frequently used interchangeably or ambiguously in the context of HFS. For instance, learners may describe experiencing anxiety or discomfort during emotionally charged scenarios, but the threshold at which such reactions constitute harm is infrequently defined [39, 47, 48]. While certain levels of stress may enhance alertness and performance [22], high levels of improperly managed anxiety can lead to emotional distress and disengagement from learning [16, 19]. Burnout, typically associated with long‐term occupational exhaustion, has been coupled with psychological harm in cases where learners are repeatedly exposed to emotionally draining simulation experiences that increase allostatic load, a term referring to the cumulative physiological burden placed on the body as it attempts to adapt to repeated or chronic stressors [49]. Psychological harm may also arise when simulation scenarios or debriefing practices unintentionally conflict with a learner’s personal values or beliefs [50], creating conditions that may contribute to moral injury. Perhaps most commonly, a loss of psychological safety is used as shorthand for most emotionally unsafe learning conditions [29, 51, 52], despite its inability to capture the full depth of emotional disruption implied by psychological harm.

#### 4.1.1. Psychological Harm Across Disciplines

While many terms overlap with the construct of psychological harm, multiple disciplinary uses of the term psychological harm also exist. Within the context of healthcare professionals, such as nurses, psychological harm refers to any injury impacting the mental, emotional, or social domain that is related to work [53, 54]. Trauma stemming from workplace violence or burnout is often a precipitating factor for psychological harm among healthcare workers [55]. When left unaddressed, such harm can impact not only the healthcare workers’ well‐being but also the quality of care delivered to patients [56]. From a patient perspective, psychological harm can arise when emotionally damaging healthcare interactions compromise trust, dignity, or a sense of personal worth [57].

Beyond healthcare education, psychological harm is conceptualized as a sustained injury to psychological well‐being arising from traumatic or chronically adverse conditions [58, 59]. Across disciplines, it denotes enduring disruption to identity, coping capacity, and emotional functioning rather than transient distress [60, 61]. This framing situates psychological harm as a form of lasting psychological injury that extends beyond momentary discomfort and reflects deeper impairment [62–64].

#### 4.1.2. Psychological Harm in HFS

In HFS, psychological harm is used less formally and often inconsistently [26, 65]. Most commonly, simulationists refer to related constructs such as emotional distress [66–72] or a loss of psychological safety [29, 46, 47, 51, 52] when describing a learner’s response to high‐stakes or emotionally charged scenarios. While HFS is generally designed to promote experiential learning, it can also provoke intense emotional reactions, particularly when involving simulated death [71, 73, 74] or deception [70, 71, 75] without adequate preparation or debriefing [66, 71]. Learners have described experiences of being overwhelmed [26, 38, 71] or emotionally unsafe [51], which resembles the psychological harm documented in clinical settings [57]. This suggests that protecting learners and healthcare professionals from emotional harm is not only an ethical concern in clinical practice but also a responsibility of educational settings, especially in psychologically demanding HFS.

Recently, Harvey et al. [25] explored this ambiguity through a hermeneutic study of undergraduate nursing students, revealing that psychological harm in simulation is often experienced as deeply personal, shaped by perceived failure, relational dynamics, and lasting self‐doubt. Participants described harm not just as transient emotional distress but as a disruption to their self‐worth and future engagement in clinical or simulation learning. These findings reinforce that psychological harm in HFS is not simply a by‐product of intense emotions but a complex, subjective outcome influenced by how learners internalize their experiences [25].

Across contexts, psychological harm consistently refers to emotional injury that disrupts an individual’s sense of well‐being. Through a constructivist lens, psychological harm in HFS is understood as a subjective and situated phenomenon, one that emerges when learners are unable to reconcile the cognitive, emotional, or relational demands of the simulation. Importantly, however, psychological harm is rarely caused by the scenario alone; it is most often a consequence of how the simulation is facilitated and the interpersonal dynamics established by the simulationist. Recent work has documented instances where unclear expectations, punitive reactions, or incivility from untrained or unaware facilitators create conditions that violate psychological safety and produce lasting distress [31]. Thus, psychological harm is shaped not only by the scenario and the learner’s interpretation but also by the quality of facilitation and adherence to trauma‐informed, psychologically safe practices.

Together, these insights support a broader and clearer conceptualization: Psychological harm in HFS can be described as a sustained or intense emotional disruption resulting from simulation‐based learning that exceeds expected stress and impairs the learner’s ability to engage, reflect, or feel safe within the educational environment.

### 4.2. Defining Attributes

In alignment with Step 4 of Walker and Avant’s [24] method, defining attributes, the core characteristics most frequently associated with a concept and essential for its existence, were identified to distinguish psychological harm in HFS from related constructs. These attributes represent the most consistent themes found across various uses of the term and help distinguish the concept from similar or related phenomena. Defining attributes that are associated with psychological harm in HFS include an absence of psychological safety, emotional disruption that exceeds the expected learning stress, and a sustained emotional impact beyond simulation. Table 1 presents a summary of psychological harm in HFS, including its defining attributes, antecedents, and consequences.

**TABLE 1:** Summary of defining attributes, antecedents, and consequences of psychological harm in high‐fidelity simulation.

Concept	Defining attributes	Antecedents	Consequences
Psychological harm in high‐fidelity simulation	• Absence of psychological safety • Emotional disruption exceeding expected learning stress • Sustained emotional impact	• Emotionally intense or ethically challenging scenarios • Insufficient cognitive or emotional preparation • Unskilled or harmful facilitation practices • Learning environments that prioritize performance	• Diminished self‐confidence • Disengagement or avoidance of simulation • Impaired transfer of learning to clinical practice

#### 4.2.1. The Absence of Psychological Safety

As revealed by the literature, a central attribute of psychological harm in HFS is the absence of psychological safety [29, 30, 46, 47, 51, 52, 66, 70]. Psychological safety, commonly understood as the belief that one can express themselves and speak openly without fear of humiliation or reprisal, is essential for fostering effective simulation environments [29, 30, 46, 47]. The absence of psychological safety within HFS may transform typical learning stress into psychologically harmful experiences [51, 52]. Unsafe or unskilled facilitation is a common source of this loss of psychological safety, with facilitator behaviors, rather than the scenario itself, often shaping whether a simulation becomes emotionally harmful [31]. Trauma‐informed and empathetic prebriefing practices are especially critical in these situations, as they help rebuild psychological safety when learners have previously encountered unskilled, inattentive, or punitive facilitation. Learners who perceive themselves as overly scrutinized or unsupported may withdraw, suppress emotional reactions, or internalize perceived failures [30, 47, 51, 67, 70]. When learners do not believe that they can make mistakes without facing consequences, the simulation experience can evoke excessive emotional distress [46, 52]. Structured and empathetic prebriefings are key to establishing clear prebriefing agreements that support psychological safety and outline expectations for respectful, collaborative engagement [29, 47]. However, when prebriefing is absent, learners may feel unprepared and perceive that mistakes could negatively impact their reputation or professional standing [46]. Likewise, debriefings that lack empathy, structure, or opportunities for critical reflection may leave learners feeling vulnerable or ashamed, particularly if they believe they made a mistake or performed inadequately [25, 47, 66].

### 4.3. Emotional Disruption That Exceeds the Expected Stress of Learning

Eustress in HFS is both expected and necessary [22, 35]. It can enhance realism, promote critical thinking, and prepare learners for emotionally charged clinical environments [20, 23, 29]. Within this context, moderate levels of stress are anticipated and can provide pedagogical benefits. However, stress may escalate beyond what learners can regulate or recover from within the safe boundaries of the educational setting [76–79], in which case psychological harm arises. Indicators of excessive emotional disruption include sustained anxiety [16, 47], crying [41], or emotional withdrawal [15, 33], especially when these features persist after simulation and interfere with reflection or engagement [26, 66, 67]. This level of distress often reflects a mismatch between the learner’s coping capacity and the demands of the scenario, particularly when the simulation involves heightened emotional intensity or an unskilled facilitator [31, 68, 70, 73, 75]. In contrast to the expected stress that typically resolves through debriefing and reflective processing, emotional disruption associated with psychological harm may linger, impair learning, and evoke feelings of shame, helplessness, or failure among learners [25, 26, 33, 47].

#### 4.3.1. Sustained Emotional Impact

A sustained emotional impact that extends beyond simulation marks an identifiable point between the expected stress that accompanies simulation and psychological harm in HFS. While temporary emotional activation is anticipated and often useful [73, 77], these responses are typically self‐limiting and resolve during structured debriefing with a skilled facilitator [28, 35, 39, 52, 67]. In contrast, some learners may experience a prolonged emotional impact from HFS. Even when scenarios evoke unexpected or intense emotions, the facilitator bears responsibility for monitoring distress, providing psychological support, and ensuring that emotional activation does not escalate into harm [10]. They may carry this distress into clinical placements, question their professional identity, or develop persistent avoidance of simulation altogether [15, 22, 25, 33]. Additionally, physiological stress responses across repeated simulation exposures suggest that learners may experience sustained psychobiological strain if adequate recovery is not supported [49]. Such reactions suggest that the simulation has imprinted itself in a way that exceeds the intended scope of educational discomfort, indicating psychological harm [26, 33]. This prolonged sensitivity often reflects a dysregulation of emotional processing initially provoked by a learning environment that failed to adequately buffer the learner’s emotional response [26, 71]. Scenarios involving moral distress, death, or deception may intensify this reaction, particularly when learners struggle to reconcile their emotional experience with their professional role [70–72]. Traditional debriefing methods often prioritize learning outcomes over emotional processing, which may leave distress unresolved and contribute to lasting psychological effects [20]. As this emotional impact endures, it may interfere with learner’s ability to reengage in clinical learning, disrupt their confidence, and negatively influence how they view themselves within the healthcare profession [33].

### 4.4. Constructed Cases

Consistent with Steps 5 and 6 outlined by Walker and Avant [24], constructed cases were developed as illustrative examples used to clarify and refine the boundaries of a concept by demonstrating the presence or absence of its defining attributes. These cases serve to illuminate how a concept operates across varying contexts and degrees of alignment.

#### 4.4.1. Model Case

Walker and Avant [24] describe a model case as a constructed example that illustrates the full expression of a concept by including all of its defining attributes. The following scenario represents a clear instance of psychological harm in HFS, demonstrating the absence of psychological safety, emotional disruption that exceeds the expected stress of learning, and a sustained emotional impact:

During a high‐fidelity pediatric code simulation, a third‐year nursing student (Learner A) is assigned the team leader role, despite receiving minimal preparation about the scenario’s intensity or what would be expected of them. The prebriefing had been brief and procedural, offering little emotional grounding or encouragement, and Learner A entered the room still unsure of whether it was acceptable to make mistakes or ask for help. As the scenario unfolded, the infant mannequin began to deteriorate rapidly, a planned cardiac arrest that had not been mentioned beforehand. Learner A felt their chest tighten as unfamiliar faculty stood behind a one‐way mirror, observing silently and taking notes. No facilitator intervened to clarify expectations or acknowledge the growing tension, and Learner A interpreted the silence as judgment. Their hands shook as they issued orders; with each misstep, the facilitator’s raised eyebrow and clipped tone made them fear they were failing publicly. When the infant ultimately “died,” the room fell quiet. Instead of pausing to check in with the group or acknowledge the emotional weight of the event, the debrief, led by an unskilled facilitator, began immediately with a focus on clinical errors. Comments such as “You hesitated too long here” and “This is why the infant didn’t make it” were delivered without context or reassurance, leaving Learner A feeling personally responsible for the outcome. At no point were their reactions, fears, or stress responses explored. Learner A left the simulation trembling, believing that they had performed incompetently and that their instructors now questioned their suitability for pediatric care. Over the following weeks, they avoided volunteer opportunities in simulation, experienced anxiety when caring for infants during clinical rotations, and found themselves replaying the scenario with a sense of shame and dread.

#### 4.4.2. Borderline Case

A borderline case includes some, but not all, of a concept’s defining attributes, offering a partial example of the phenomenon [24]. It serves to clarify the boundaries of the concept by illustrating situations that resemble the concept but fall short of fully meeting its criteria. Thus, borderline cases help differentiate essential attributes from those that may be present but are not central to the concept’s definition. In the context of psychological harm in HFS, an example of a borderline case is as follows.

A second‐year nursing student (Learner B) arrives for a high‐fidelity respiratory failure simulation feeling mildly anxious but prepared to contribute. That sense of readiness fades the moment they step into the simulation room. The equipment layout is unfamiliar, no orientation has been provided, and their assigned role, to manage oxygen therapy and communicate findings, has not been discussed in detail. Faculty and peers watch silently from behind a one‐way mirror, and Learner B can feel their presence before even seeing them, a prickle at the back of their neck that sharpens their awareness of every movement they make. The scenario escalates quickly. The patient’s breathing becomes labored, alarms sound, and the team leader demands a rapid update on oxygen saturation. Learner B reaches for the flowmeter but hesitates, unsure whether they are adjusting it correctly. Their mind blanks. When the team leader asks for vital signs, they open their mouth but no words come out. The brief freeze feels like an eternity, and Learner B’s face grows hot as they imagine the observers cataloging their hesitation. During the debriefing, a poorly trained facilitator highlights what the group “should have done sooner,” emphasizing delayed interventions without asking how the learners experienced the moment. No space is offered for emotions, uncertainty, or processing. Learner B leaves feeling embarrassed, quietly replaying the freeze, convinced it exposed their lack of preparedness. Over the next several days, they catch themselves ruminating about the scenario, feeling a knot of anxiety whenever they think about future simulations. But when their clinical instructor checks in later that week, Learner B shares their worry. The instructor validates the experience, reframes the freeze as a common part of early learning, and helps them understand what went well. Learner B exhales in relief. By the next simulation session, they return with renewed confidence, participate fully, and feel grounded rather than fearful.

In this case, Learner B demonstrates two defining attributes of psychological harm in HFS: an absence of psychological safety and emotional disruption that exceeds the expected stress of learning. The lack of orientation, the pressure of being observed, and a judgment‐focused debriefing collectively erode their sense of safety and heighten their emotional response. However, the key distinguishing feature is the lack of a sustained emotional impact. Because Learner B receives timely support, can process the experience, and reengages in simulation with restored confidence, the emotional disturbance does not persist. This example illustrates how psychologically unsafe moments can emerge in simulation yet remain borderline when appropriate intervention prevents them from becoming enduring psychological harm.

#### 4.4.3. Related Case

Walker and Avant [24] identify a related case as a situation that is connected to the phenomenon of interest but does not contain its defining attributes. Its purpose is to demonstrate how a concept may be confused with adjacent experiences that share contextual or emotional similarities while remaining theoretically distinct. An example of a related case is as follows.

During a high‐fidelity postpartum hemorrhage simulation, a third‐year nursing student (Learner C) enters the room with a focused calm. They have practiced the postpartum assessment many times, but as the scenario begins and the mannequin’s blood pressure drops, a sudden realization hits them, and they cannot recall the exact sequence for reassessing an unstable postpartum patient. Their pulse quickens. The room feels warm. A brief wave of embarrassment washes over them as they imagine their peers behind the glass noting their hesitation. The team leader asks for an update, and Learner C pauses, taking a moment to gather themself. Before panic can take hold, one of the facilitators steps a little closer, softening their voice as they offer a gentle prompt: “Start with what you know. You’ve got this.” The reassurance grounds them. Learner C nods, reassesses the patient, and delivers the information clearly. The scenario moves forward smoothly. In the debriefing, the facilitators maintain the same steady, supportive tone. Rather than highlighting mistakes, they ask Learner C what cues they noticed, how they decided what to prioritize, and what felt challenging in the moment. Learner C admits they froze briefly and felt embarrassed, but the facilitators normalize the reaction and emphasize how early recognition of uncertainty is part of developing clinical reasoning. As the conversation unfolds, Learner C begins to see the scenario not as a failure but as an opportunity. By the end of the debrief, they laugh lightly at their initial worry and identify concrete steps for improving their postpartum assessment skills. When they leave the room, they feel more confident, almost energized, about managing postpartum emergencies in the future.

This case demonstrates emotional activation within the simulation context but does not meet the defining attributes of psychological harm. Learner C experiences acute stress and momentary uncertainty, which are expected and pedagogically useful responses in simulation‐based learning. Importantly, psychological safety is preserved through a calm, supportive facilitator and a reflective debriefing that acknowledges, normalizes, and contains their emotional response. Because Learner C can process the experience, integrate the feedback, and leave with increased confidence, the emotional activation remains transient rather than harmful. This case illustrates the distinction between normative performance‐related stress and psychological harm.

#### 4.4.4. Contrary Case

A contrary case is a constructed example that does not contain any of the defining attributes of the concept, serving as a clear illustration of what the concept is not [24]. In the context of HFS, a situation that does not provoke psychological harm is as follows.

A first‐year nursing student (Learner D) arrives early for the HFS on postoperative complications, their nerves buzzing with the familiar combination of excitement and uncertainty. Before the session begins, the highly skilled facilitators welcome the group into the simulation suite, walking them through the equipment, layout, and expected actions step by step. They remind the students that the session is formative, not evaluative, and emphasize that mistakes are expected, encouraged, and essential for learning. The tone is warm, unrushed, and genuinely reassuring, and Learner D feels their shoulders relax as they settle into their assigned role. When the scenario begins and the patient’s oxygen saturation dips unexpectedly, Learner D pauses, momentarily unsure of the appropriate next step. Before anxiety can build, their teammates respond collaboratively, offering suggestions and checking in with them. The facilitator, whose voice Learner D recognizes from earlier labs, steps closer and gently prompts, “Walk us through what you’re thinking, take your time.” The calmness in their tone steadies them, and Learner D proceeds confidently, adjusting the oxygen delivery and communicating their assessment to the team leader. The debrief takes place in a small, comfortable room where the trained facilitators first invite students to explore their emotional reactions to the scenario before shifting into a discussion of clinical decision‐making, reflecting the standard that emotional processing precedes cognitive reflection. They emphasize confidentiality, normalize moments of hesitation, and highlight each learner’s strengths. Learner D shares that they felt briefly nervous when the patient’s saturations dropped, and the facilitator nods, validating the reaction and reframing it as a natural early‐practice response. By the end of the session, Learner D feels challenged but supported, energized even, as they realize the experience has strengthened their confidence for their upcoming hospital rotation.

In this case, none of the defining attributes of psychological harm are present. The simulation environment is intentionally structured to promote psychological safety through a detailed prebriefing, familiar and supportive facilitators, and a formative, nonevaluative framing. Although the scenario includes a clinical challenge, Learner D receives consistent emotional support, clear expectations, and opportunities to reflect without fear of judgment. This case demonstrates how protective factors, such as transparent communication, empathetic facilitation, and reflective debriefing, create conditions that bolster learner confidence and prevent psychological harm.

### 4.5. Antecedents

In accordance with Step 7 of Walker and Avant’s [24] method, antecedents are conditions or events that must be present prior to the occurrence of a concept. Antecedents identified for psychological harm in HFS include the exposure to simulation content with high emotional or ethical intensity, insufficient cognitive or emotional preparation for simulation, unskilled or harm‐inducing facilitation practices, and learning environments that prioritize performance outcomes over psychological safety. See Table 2 for a summary of the contributing and protective factors for psychological harm in HFS.

**TABLE 2:** Contributing and protective factors related to psychological harm in HFS.

Contributing factors	Protective factors
‐ Unexpected death, failure, or emotionally charged scenarios without adequate prebriefing ‐ Use of deception or lack of informed consent for emotionally intense scenarios ‐ High‐stakes evaluation or perceived performance assessment ‐ Being observed, filmed, or publicly critiqued without a psychological safe structure ‐ Lack of trust, unfamiliar facilitators, or strained learner–facilitator relationships ‐ Unskilled, untrained, or malignant facilitators ‐ Judgmental, critical, or overly mistake‐focused debriefing ‐ Hierarchical dynamics that silence learners ‐ Learners internalize mistakes as evidence of incompetence ‐ Learners are “thrown in” without orientation, objectives, or time expectations ‐ Emotionally triggering content without adequate learner preparation ‐ Learner confidentiality is compromised during observation or feedback	‐ Clear, honest prebriefing that prepares learners for emotionally and ethically complex content ‐ Clear orientation, structured objectives, and communicated expectations before simulation—Framing simulation as a formative learning experience rather than a summative assessment ‐ Small‐group debriefings emphasizing support and confidentiality ‐ Facilitators building rapport and familiarity with learners to foster trust and reduce power imbalance ‐ Trauma‐informed or psychological safe debriefing models to validate learner experiences ‐ Intentional flattening of hierarchies to support open dialog and interprofessional respect ‐ Facilitators normalize error and reframe mistakes as essential components of professional learning ‐ Advance disclosure of sensitive content and optional opt‐out without penalty

#### 4.5.1. Simulation Content With High Emotional or Ethical Intensity

Simulation scenarios that carry significant emotional or ethical weight, such as patient death, delivering bad news, or navigating moral dilemmas, do not, in themselves, constitute harm but rather heighten a learner’s emotional vulnerability [70, 71, 73]. When learners are immersed in scenarios that challenge their values, sense of responsibility, and emotional regulation, they become more susceptible to psychological distress if appropriate supports are not in place [25, 26]. This heightened emotional or ethical load amplifies the risk of psychological harm, particularly when the learner lacks adequate preparation or opportunities for transformative reflection [20, 47]. Rather than being inherently harmful, these scenarios function as priming conditions, increasing the emotional salience of the experience and thereby creating conditions under which harm may arise [22, 25].

#### 4.5.2. Insufficient Cognitive or Emotional Preparation for Simulation

When learners enter simulation without a clear understanding of the objectives, expectations, or equipment, they are more likely to experience uncertainty or a heightened level of stress during the scenario [20, 47, 52]. This lack of cognitive readiness may impair performance, which in turn can trigger feelings of inadequacy or perceived failure [25, 46, 78]. Insufficient emotional preparation, such as inadequate prebriefing on the potential emotional demands of the scenario or a failure to normalize emotional responses, can leave learners vulnerable to distress and overwhelmed by the simulation experience [20, 25, 26]. Without appropriate framing, well‐designed simulations may feel disorienting or punitive to some learners [29, 71]. As a result, learners become more susceptible to interpreting mistakes as personal deficits rather than opportunities for growth, increasing the risk of internalized shame and emotional withdrawal [25, 51, 70]. Inadequate preparation creates vulnerability, reducing resilience when learners are faced with complex or emotionally charged simulation experiences, ultimately providing the foundation for psychological harm. Inadequate facilitator preparation or lack of training in trauma‐informed or psychologically safe practices further amplifies these risks, as unskilled facilitation can unintentionally intensify learner distress [10, 32].

#### 4.5.3. Unskilled or Harm‐Inducing Facilitation Practices

Numerous studies demonstrate that facilitator behaviors, not the scenario itself, are often the primary source of psychological distress or harm [25, 29]. When simulationists lack training in prebriefing, relational communication, or debriefing frameworks, or worse, demonstrate punitive, shaming, or judgmental behaviors, the environment shifts from psychologically safe to psychologically threatening [29, 31]. Learners frequently attribute their distress to the facilitator’s tone, approach, and interpersonal conduct, rather than the clinical content or fidelity of the simulation. Untrained or unskilled facilitators disrupt trust, undermine learner confidence, and increase the likelihood that mistakes are interpreted as personal inadequacy rather than opportunities for learning [25, 29, 47]. Malignant or harmful facilitation practices, such as humiliation, sarcasm, withholding guidance, inconsistent expectations, or role ambiguity (evaluator vs. educator), have been explicitly identified as contributors to psychological harm [25, 26, 31]. These behaviors amplify learner anxiety, elevate performance pressure, and erode the foundational trust essential for psychological safety. Even when simulationists believe they have created a “safe container,” it is the learner’s perception, shaped by facilitator demeanor, history, and interpersonal dynamics, that ultimately determines whether safety has been achieved [25, 29].

#### 4.5.4. Learning Environments That Prioritize Performance

When simulation is framed as a space to demonstrate competence rather than foster growth, learners may experience heightened anxiety and self‐monitoring, which can inhibit participation and reflection. The dual role of instructors as both evaluators and facilitators can further amplify this tension, fostering a fear of judgment among learners [25]. Additionally, debriefing models that emphasize performance outcomes over emotional processing may leave distress unaddressed, shifting the simulation from a learning opportunity to a perceived test [15, 20]. Learners that feel as if they are being evaluated rather than supported may become reluctant to take risks or disclose uncertainty. If these feelings persist, psychological safety may erode, and the learners are more likely to internalize mistakes as personal failure, contributing to a psychologically harmful environment [25, 26, 33].

### 4.6. Consequences

Consequences are the outcomes that occur as a result of the concept’s presence [24]. Identifying consequences surrounding psychological harm in HFS provides insights and practical significance by revealing the potential impact on learners. Consequences that result from psychological harm in HFS include diminished self‐confidence, avoidance, and disruption in the transfer of learning to clinical practice.

#### 4.6.1. Diminished Self‐Confidence

Diminished self‐confidence arises when psychological harm leads learners to interpret their simulation performance as a reflection of personal inadequacy rather than an opportunity for growth [25, 67]. In these moments, distress undermines the learner’s sense of capability and belonging, shaping how they perceive themselves within the professional role [26, 67]. This internal shift in self‐perception, even when not outwardly visible, can have a lasting effect on confidence, self‐efficacy, and the learner’s belief in their potential to succeed as a healthcare professional [18, 25, 33, 35].

#### 4.6.2. Avoidance

Rather than engaging constructively with future learning opportunities, learners who experience psychological harm may distance themselves, adopting passive roles, suppressing emotional responses, or avoiding simulation altogether [21, 25]. This avoidance serves as a self‐protective strategy, shielding learners from further emotional injury or judgment. Over time, such withdrawal not only limits the learner’s access to key experiential learning but also devalues their sense of belonging within the simulation space [26, 33, 52, 70].

#### 4.6.3. Disruption in the Transfer of Learning to Clinical Practice

Psychological harm can interfere with a learner’s ability to carry knowledge and skills forward into clinical practice [21, 25, 33]. Rather than emerging from the simulation with readiness, harmed learners may feel hesitant or disengaged from the learning process [52, 70]. This disruption is rarely momentary but rather lingers, subtly eroding confidence and prompting learners to question their clinical judgment. Over time, the intended bridge from theory to practice is weakened, and opportunities for learners to gain clinical competence are minimized [15, 19, 25, 26, 35, 76].

### 4.7. Empirical Referents

As outlined in Step 8 by Walker and Avant [24], empirical referents are measurable indicators that demonstrate the existence of a concept. Although closely aligned with defining attributes, empirical referents serve to operationalize the concept by offering observable ways it can be detected and evaluated. In the case of psychological harm in HFS, the referents identified reflect the emotional, physiological, and behavioral traces left behind when harm has occurred.

#### 4.7.1. Self‐Reported Distress

Psychological harm often reveals itself through the learner’s own words during or after simulation. Expressions of shame or inadequacy, often during debriefing, offer direct insights into the internal disruption caused by the simulation experience [25, 26, 33, 47, 52].

#### 4.7.2. Physiological Markers

Emotional injury during simulation can activate the body’s stress response, producing measurable changes such as increased cortisol or anxiety [35, 49, 72]. When these physiological shifts occur in tandem with cognitive disruption or emotional withdrawal, it provides objective evidence that the simulation exceeded the learner’s adaptive capacity [19, 25, 35, 41, 72].

#### 4.7.3. Behavioral Indicators

Learners who experience psychological harm may exhibit behavioral changes that communicate an emotional overload. In some cases, this harm becomes visible when a student freezes, withdraws, or flees from the simulation environment, responses that are not common in routine anxiety but rather demonstrate overwhelming distress. Reactions may also include crying, hyperventilation, panic attacks, and the need to physically leave the space to regain composure [25, 41, 72]. When simulation is perceived as emotionally unsafe or retraumatizing, these outward behaviors may reflect an internal effort to self‐protect, signaling the presence of psychological harm.

## 5. Discussion

This concept analysis sought to clarify the meaning of psychological harm in HFS, a phenomenon that remains inconsistently defined and often conflated with expected stress or emotional discomfort in nursing education. Guided by the framework established by Walker and Avant [24], this analysis revealed that psychological harm in HFS is a multifaceted, learner‐dependent construct, characterized by an absence of psychological safety, emotional disruption exceeding expected stress, and a sustained impact that interferes with learning or clinical practice. This concept analysis advances definitional clarity by distinguishing psychological harm from normative stress responses, establishing a foundation for future operationalization and measurement in nursing simulation research.

While simulation is designed to challenge learners and mimic the complexities of clinical care, findings from this concept analysis underscore that not all emotional stress is productive. Appropriate levels of stress can enhance realism and promote cognitive engagement [8, 22, 33]. However, when psychological safety is compromised and learners are unsupported in emotionally intense or ethically complex scenarios, simulation may exceed a learner’s adaptive threshold, leading to emotional withdrawal, shame, or disengagement [26, 72].

Recently, Harvey et al. [25] have deepened the understanding of this concept by demonstrating how psychological harm is internalized. Participants in their study described simulation experiences that undermined self‐worth, invoked feelings of failure, and altered future engagement in both simulation and clinical environments, reflecting a fundamental injury to the learner’s sense of competence and belonging. This aligns with broader educational theory suggesting that emotional injury, when left unprocessed, can impair learning, promote avoidance, and compromise self‐efficacy [18, 67].

A key finding of this analysis is the critical role of relational dynamics, particularly with simulationists and peers, in moderating psychological harm. These relational dynamics are profoundly influenced by facilitator competence; skilled simulationists are uniquely positioned to anticipate, detect, and mitigate learner distress during prebriefing, facilitation, and debriefing [10]. Facilitator behaviors perceived as punitive or evaluative can heighten feelings of vulnerability. In contrast, transparent communication, familiarity, and empathy appear to mitigate harm [32]. Additionally, peer support emerged as a protective mechanism against psychological harm. Learners who experienced distress reported “trauma bonding” with their peers, using humor and solidarity as coping strategies [25]. This reinforces that psychological harm is not only shaped by the scenario itself but also by the relational and environmental context in which learning occurs.

These findings align with the HSSOBP [10], which identify skilled facilitation, structured prebriefing, and reflective debriefing as central to maintaining psychological safety. The standards, echoed by recent work on student abuse in simulation [31], emphasize that psychological harm rarely arises from the simulation activity itself but from how the experience is facilitated, framed, and debriefed. In this context, harm represents a breakdown in facilitation practice rather than an inherent property of simulation design. Integrating trauma‐informed and emotionally attuned facilitation strategies may therefore represent the most effective approach to preventing psychological harm and promoting learner well‐being within simulation‐based education [20].

From a pedagogical standpoint, this analysis highlights the importance of trauma‐informed simulation design. As recommended by Harvey and Carter‐Snell [80], leveraging a highly trained facilitator to apply trauma‐informed principles such as anticipating emotional responses, providing choice and control, and prioritizing emotional processing in debriefs, may help prevent the onset or escalation of harm. Similarly, El Hussein et al. [81] advocate for the consistent use of structured prebriefing to foster psychological safety, clarify expectations, and mitigate stress, supporting learners’ emotional well‐being before the HFS begins. Learners may also benefit from clear role expectations, emotionally safe prebriefings, and structured opportunities to express distress without judgment [28, 39].

The consequences identified in this analysis, diminished self‐confidence, avoidance, and impaired clinical transfer, reinforce the long‐term educational and professional risks of psychological harm. These effects challenge the assumption that simulation is inherently safe and suggest that emotional outcomes must be monitored alongside skill acquisition. When learners associate simulation with fear or inadequacy, their ability to participate meaningfully in future learning is jeopardized. Although existing standards outline how skilled prebriefing, facilitation, and debriefing can prevent psychological harm [10], evidence suggests that implementation remains inconsistent. Many facilitators receive limited preparation in trauma‐informed or emotionally attuned simulation practices, which can leave learners vulnerable despite well‐designed scenarios. Addressing this gap requires not only reinforcing adherence to existing standards but also developing educational strategies that enhance facilitator self‐awareness, emotional intelligence, and responsiveness to learner distress.

A growing concern in contemporary simulation practice is the increasing use of simulation for high‐stakes competency assessments. More programs than ever before are adopting simulation as an evaluative tool, often without ensuring that faculty are adequately trained in facilitation, prebriefing, or debriefing practices. When psychological safety is not intentionally established, performance‐driven assessment environments can amplify anxiety, suppress learner autonomy, and heighten the risk of psychological harm, particularly for novice learners. This trend underscores the critical need for institutions to prioritize facilitator training and align assessment‐based simulation with the HSSOBP [10] to prevent harmful learning environments.

This analysis also highlights the risk of overextending the concept of psychological harm in ways that could restrict educational innovation or limit the creative use of challenging, high‐fidelity scenarios. A clear, evidence‐informed definition helps guard against misuse, ensuring that concerns about psychological harm do not discourage appropriate levels of challenge or the development of complex, realistic simulations that foster deep learning.

### 5.1. Future Research

While this concept analysis offers foundational clarification on psychological harm in HFS, it also reveals several key gaps in the literature that warrant further research. Although the HSSOBP [10] provide a robust framework for preventing psychological harm through effective prebriefing, facilitation, and debriefing, inconsistent application of these standards remains a challenge. Future research should therefore focus on developing and evaluating facilitator‐support frameworks, training interventions, or observational strategies that promote early recognition and mitigation of learner distress in real time. Such work would strengthen the capacity of facilitators to apply trauma‐informed principles consistently, complement existing standards, and ensure emotional safety across diverse simulation contexts.

Future work could also explore proactive approaches to identifying learner readiness and emotional vulnerability before engaging in simulation‐based learning experiences. Rather than relying on formal measurement tools, facilitators can incorporate intentional dialog during prebriefing to understand learners’ prior simulation experiences, perceived challenges, and emotional readiness. Open discussion before the scenario allows facilitators to anticipate potential triggers, normalize stress responses, and tailor psychological support as needed. As simulation itself is intended to provide a safe, supportive environment for learning, early recognition of emotional needs could help ensure that the experience continues to protect rather than inadvertently distress learners, particularly those encountering simulation for the first time or transitioning into new clinical roles.

Longitudinal studies are also needed to explore how psychological harm influences learner outcomes over time, including confidence, professional identity, and engagement in future simulation. Cross‐cultural inquiry is also needed to examine how norms surrounding communication, hierarchy, authority, and emotional expression shape learners’ vulnerability to psychological harm. Because simulation practices and expectations differ across cultural and institutional contexts, such work is essential to determine whether the defining attributes identified in this analysis hold universal relevance or require cultural adaptation.

### 5.2. Limitations

This concept analysis was limited to peer‐reviewed literature published in English between 2010 and 2025, which may have excluded relevant studies published outside this timeframe or in other languages. Although efforts were made to capture diverse perspectives on psychological harm, the available literature disproportionately reflects the experience of undergraduate nursing students in North America and European contexts. As such, the findings may not fully represent simulation experiences in different cultural, institutional, or professional settings, such as medicine, allied health, or international nursing education programs. Additionally, this analysis focused exclusively on HFS; other simulation modalities, such as low‐fidelity, virtual reality, or screen‐based formats, were excluded and may present distinct emotional risks that were not captured.

## 6. Conclusion

As HFS becomes increasingly central to nursing education, so too does the responsibility to ensure it does not unintentionally harm the learners it seeks to empower. To our knowledge, this analysis offers one of the first comprehensive, literature‐based conceptualizations of psychological harm in HFS, distinguishing it from expected stress and clarifying its defining attributes: the absence of psychological safety, emotional disruption that exceeds the expected stress of learning, and a sustained impact that extends beyond the simulation experience. By framing psychological harm as a learner‐centered and context‐dependent phenomenon, this analysis challenges the assumption that all emotional reactions in simulation are pedagogically productive. It highlights the need for trauma‐informed design, emotionally aware facilitation, and greater accountability for learner well‐being. At the same time, clear conceptual boundaries can help ensure that the term psychological harm is not misapplied in ways that constrain educational creativity, limit appropriate challenge, or deter innovation in simulation design. These findings not only equip facilitators with a clearer lens through which to recognize and prevent harm but also lay the groundwork for future empirical research and simulation standards that prioritize both skill acquisition and psychological integrity. If simulation is to remain a transformative tool in healthcare education, it must also be a safe one.

## Funding

This work was supported by Alberta Innovates—Health Solutions.

## Conflicts of Interest

The authors declare no conflicts of interest.

## Data Availability

The data that support the findings of this study are available from the corresponding author upon reasonable request.
